# Influence of IL-20 on lumbar disc degeneration:An experimental study

**DOI:** 10.12669/pjms.311.5720

**Published:** 2015

**Authors:** Tianjing Yang, Huaqing Xu

**Affiliations:** 1 Tianjing Yang, Department of Pathology, Tianjin 4th Centre Hospital, Tianjin, 300140, China.; 2 Huaqing Xu, Department of Surgery, Tianjin 4th Centre Hospital, Tianjin, 300140, China.

**Keywords:** IL-20, Lumbar degeneration, Modified Pfirrmann Grading System, Proteoglycan

## Abstract

**Objective:**

To determine the influence of IL-20 on the development of lumbar degeneration.

**Methods:**

The study design was prospective and carried out in Tianjin Fourth center Hospital, Tianjin, China between Jan 2012 and Jan 2014. Sixty-nine patients with degenerative disc disease treated surgically were included in experimental group, and fifteen patients with normal discs were included in control group. The evaluation of disc degeneration was performed using T2-weighted sagittal MRI according to the Modified Pfirrmann Grading System. After surgery, the intervertebral disc in both groups was collected and the content of proteoglycan and IL-20 were measured, the correlation between the content of IL-20, proteoglycan and the degeneration grade of lumbar disc was analyzed.

**Results:**

Compared to control group, the content of proteoglycan in experimental group is significantly lower (P=0.000), but IL-20 is significantly higher (P=0.001). In addition, with the advance of intervertebral disc degeneration, the content of IL-20 increase, while proteoglycan decrease gradually. There is significant correlation between the content of proteoglycan (p=0.001), IL-20 (p=0.002) and the degeneration grade of lumbar disc.

**Conclusion:**

In patients with degenerative disc disease, the content of IL-20 and proteoglycan has significant correlation with degeneration grade of lumbar disc, and IL-20 may promote the degeneration of lumbar disc by affecting the synthesis of proteoglycan.

## INTRODUCTION

Low back pain is one of the most common disease in the world and about 60% to 80% of the United States population have experienced low back pain during their lives.^1^ Most of low back pain can be attributed to the degenerative disc disease (DDD). Many factors have influence on the degenerative progress of lumbar intervertebral disc. The biomechanical factors play an important role,^2^ and the abnormal stress in intervertebral disc may result in the minor injury in annulus fibrosus, inducing immune and inflammatory reaction in nucleus pulposus, and lead to the production of inflammatory factors,^3^^-^^5^ such as IL-1β, TNF-α and NO, which inhibit the synthesis of proteoglycan, and aggravate the degeneration of intervertebral disc.

Many studies have been performed on the inflammation factors in degenerative intervertebral discs. In a study on twenty consecutive patients, Huang concluded IL-20 induces proinflammatory, chemotaxtic, and matrix degradative responses in the intervertebral disc, suggesting IL-20 plays a critical role in the pathogenesis of disc herniation.^6^ In addition, IL-20 is also involved in inflammatory diseases such as psoriasis, atherosclerosis, and rheumatoid arthritis, indicating IL-20 is an important inflammation factor correlated to the chronical bone and joint disease. Subsequently, we assumed IL-20 may aggravate the degeneration of lumbar disc by affecting the synthesis of proteoglycan, and the advance of lumbar disc degeneration may result in the increased expression of IL-20. While, no correlated studies have been published in English literatures.

Therefore, we carried out a comparative investigation on the content of IL-20 and proteoglycan in lumbar disc of patients with DDD and without. The objective of this study was to detect the correlation between IL-20 and the degeneration grade of lumbar disc, analyze the influence of IL-20 on the development of intervertebral disc degeneration.

## METHODS

***Patients: ***The study was prospective and carried out in Tianjin Fourth center Hospital, Tianjin, China between Jan 2012 and Jan 2014. The patients with DDD treated surgically in spine department of our hospital during this period were included in experimental group if they met the inclusion criteria: (1) Patients with DDD; (2) The diagnosis of DDD was confirmed by symptoms, physical examination and magnetic resonance imaging (MRI). However, the patients with previous spine surgeries, concomitant scoliosis, lumbar spine fracture, lumbar instability, spondylolisthesis, spondylosis, tumors or metastatic tumors, iatrogenic abnormality, and the patients with more than one lumbar segment treated surgically were excluded.

In addition, the patients with spinal fractures treated during the same period were included in control group. To facilitate a comparative study, the participants in the control group were all young patients, which were treated surgically using anterior corpectomy. The study was approved by the ethics committee of our hospital, all the participants gave a written informed consent.

***Evaluation of the disc degeneration: ***Each participant in experimental group received MRI before surgery. The evaluation of lumbar intervertebral disc degeneration was carried out using the T2-weighted sagittal MRI according to the Modified Pfirrmann Grading System (Table-I).^7^ The grade of disc degeneration was determined by assessing the signal intensity of the intervertebral disc and disc space height. Grade 1 indicated normal disc and grade 8 the most severest degeneration.^7^

***Content of proteoglycan and IL-20: ***Immunohistochemistry was performed on the fresh specimens of nucleus pulposus following surgery. The content of proteoglycan was measured using the dimethyllmethylene blue dye binding assay.^8^ The level of IL-20 was measured by enzyme-linked immunosorbent assay using commercially available detection kits (Adiponectin: Fenyu Biotech, Shenzhen, China) according to the manufacture’s protocol.^6^

***Statistical Analysis: ***Statistical analysis was carried out using SPSS 17.0 software. The comparison of the contents of proteoglycan and IL-20 between two groups was performed using One-Way ANOVA. The correlation between the content of IL-20, proteoglycan and the grade of disc degeneration was evaluated using Spearman’s correlation analysis. A p-value of less than 0.05 was considered statistical significance.

## RESULTS

Sixty-nine patients with degenerative disc disease participated this study, which included twenty-two females and forty-seven males. The mean age was 49.8 years (ranged from 28 to 74 years). Among 69 cases, 24 were lumbar stenosis, 18 were discogenic pain and 27 were lumbar disc herniation. According to the Modified Pfirrmann Grading System, there were 11 cases in Grade 3, 16 in Grade 4, 12 in grade 5, 21 in Grade 6, 9 in Grade 7 and no case in Grade 1, Grade 2 or Grade 8. All the patients were treated surgically and the operated levels were L4-L5 in 39 cases, L5-S1in 30 cases, among which lumbar discectomy was performed in 25 cases, posterior lumbar interbody fusion in 30 cases, intervertebral disk replacement in 6 cases and transforaminal lumbar interbody fusion in 8 cases.

Fifteen patients with spinal fractures were included in the control group. There were thirteen males and two females. All the fifteen patients had magnetic resonance images, which confirm their discs were not herniated, degenerated, or otherwise injured by the fracture. The average age in the control group was 27.5 years, ranging from 19 to 31 years.

The content of IL-20 and proteoglycan in two groups was listed in Table-II. Compared to control group, the content of proteoglycan is significantly lower (P=0.00), but the content of IL-20 is significantly higher (P=0.01) in specimen group. In addition, with the advance of disc degeneration, the content of IL-20 increase, but proteoglycan decrease gradually (Table-III). The statistical analysis indicate there is significant correlation between the content of proteoglycan (p=0.001) or IL-20 (p=0.002) and the degeneration grade of lumbar intervertebral discs.

## DISCUSSION

In the present study, we detected the correlation between IL-20 and the degeneration of lumbar disc, analyze the influence of IL-20 on the development of lumbar degeneration. Up to now, no studies have been published on the subject in English literatures. 

The proteoglycan in intervertebral disc is responsible for the swelling behaviors, streaming potential, and compressive properties of the tissue, which may affect the mechanical properties of intervertebral disc.^9^ Breakdown of the large aggregating proteoglycans result in the reduction of capacity of nucleus pulposus, leading to a loss of disc hydration and decreased hydrostatic pressure. Ultimately, degeneration progresses to decreased disc height, structural changes in annulus fibrosus, annular tears and the formation of osteophytes.^10^ As a result, a loss of proteoglycans in nucleus pulposus is regarded as a clear sign of early degeneration.^9^ In the present study, compared to the control group, the content of proteoglycan in degenerative discs in experimental group decreased significantly. Moreover, the content of proteoglycan decreased with the advance of disc degeneration, and the content of proteoglycan correlated significantly with the degeneration grade of lumbar disc, indicating proteoglycan is the important sign of disc degeneration, and our results confirmed the above mentioned viewpoints.

In terms of the mechanism of intervertebral disc degeneration, inflammation mechanism was highlighted in recent years, and inflammatory factors, including interleukin, tumor necrosis factors and nitrogen monoxide, have been proved to play an important role in the process of intervertebral disc degeneration. IL-20 belongs to the IL-10 family, expressed in monocytes, epithelial, and endothelial cells, and is involved in the onset and progression of many inflammatory diseases.^11^ Tritsaris et al. found that in a rat ischemic hind-limb model, IL-20 can significantly promote reestablishment of collateral networks and blood perfusion in the ischemic skeletal muscle tissue.^12^ In a study on twenty consecutive patients with intervertebral disc herniation, Huang found that IL-20 and its receptors were detectable in human herniated disc tissues and isolated disc cells, suggesting the expression and secretion of IL-20 acts in an autocrine manner to modulate inflammatory reaction and angiogenesis, and concluded IL-20 played an important role in the pathogenesis of disc degeneration.^6^ In the current study, we found the content of IL-20 in the experimental group was significantly higher than those in the control group, which confirmed Huang’s conclusion, and subsequently, we supported the viewpoint that IL-20 play an important role in the development of intervertebral disc degeneration.

At the same time, we found with the advance of disc degeneration, the content of IL-20 increase, but proteoglycan decrease gradually, indicating IL-20 was significantly correlated with the degeneration grade of intervertebral discs. In addition, the process of IL-20 affecting the disc degeneration may be correlated closely with proteoglycan. The abnormal stress in lumbar disc level may cause the production of minor fissures, which then induce immune or inflammatory reaction and increase the contents of inflammatory factors in annulus fibrosus.^3^^-^^5^ As IL-20 is an arteriogenic cytokine^12^, in the process of disc degeneration, IL-20 may promote the vascular growth into the fissure of annulus, promote the degradation of matrix, affect the synthesis of proteoglycan and aggravate the degeneration of intervertebral disc.

**Table-I:** Modified Grading System for Lumbar Disc Degeneration

**Grade**	**Signal From Nucleus and Inner Fibers of Anulus**	**Distinction Between Inner and Outer Fibers of Anulus at Posterior Aspect of Disc**	**Disc Height**
1	Uniformly hyperintense, equal to CSF	Distinct	Normal
2	Hyperintense (>presacral fat and <CSF)±hypointense intranuclear cleft	Distinct	Normal
3	Hyperintense though < presacral fat	Distinct	Normal
4	Mildly hyperintense (slightly>outer fibers of anulus)	Indistinct	Normal
5	Hypointense (= outer fibers of anulus)	Indistinct	Normal
6	Hypointense	Indistinct	<30% reduction
7	Hypointense	Indistinct	30%–60% reduction
8	Hypointense	Indistinct	>60% reduction

**Table-II:** The content of IL-20 and proteoglycan in two groups

**Group**	**Number**	**IL-20 (pg/ml)**	**Proteoglycan (mg/g)**
Experimental group	69	62.45±9.18	56.78±11.03
Control group	15	19.26±7.86	182.45±23.91
p-value		0.001	0.000

**Table-III:** The content of IL-20 and proteoglycan in different grade of disc degeneration

**Grade**	**n**	**IL-20**	**Proteoglycan**
3	11	57.96±9.86	71.66±10.04
4	16	58.84±7.21	65.72±9.83
5	12	60.19±8.32	60.86±7.53
6	21	62.56±6.20	44.23±7.45
7	9	64.27±9.17	38.57±6.18

In conclusion, we found in this study IL-20 correlated closely with the degeneration grade of intervertebral disc in DDD patients. However, the current study has its limitation, we focused on the correlation between IL-20 and degeneration grade of lumbar discs, but its detailed mechanism and process were complicated and still unclear. Hence, more studies need to be performed in the future to find out its mechanism.

## Notes

***Conflict and interest:*** No conflict and interest declared.
